# Validity and reliability of the questionnaire of academic knowledge of teachers of basic general education

**DOI:** 10.12688/f1000research.134261.1

**Published:** 2023-06-12

**Authors:** Andrea Basantes-Andrade, Juan Carlos López-Gutiérrez, Milton Mora Grijalva, Yenney Ricardo

**Affiliations:** 1 Grupo de Investigación de Educación, Ciencia y Tecnología GIECYT, Universidad Técnica del Norte, Ibarra, Imbabura, 100105, Ecuador; 2 Grupo de Investigación Cultura, Sociedad e Imagen, Universidad Técnica del Norte, Ibarra, Imbabura, 100105, Ecuador

**Keywords:** Academic knowledge, validity, reliability, prior knowledge, questionnaire, factor analysis, Kaiser-Meyer-Olkin coefficient.

## Abstract

**Background**: The concern and analysis about the knowledge possessed by teachers of basic general education persists in academic debate and professional practice. It is noteworthy that in the studies consulted, there is no precise evidence that determines with accuracy the configurations of these knowledge factors that function as the basis of the teaching profession. Therefore, the objective of this study is to establish the construct validity and reliability of the questionnaire on the nature or origin of the academic knowledge of teachers of basic general education, adapted from the Pedagogical Content Knowledge (PCK) Competence Model.

**Methods**: A methodological study was established that applies a test to the processes of reliability and internal consistency. The construct validity was performed through (n = 8) expert judges, using Cohen’s Kappa. An exploratory factor analysis was performed following the criteria of the Kaiser-Meyer-Olkin Coefficient (KMO), the Bartlett sphericity test and the principal components extraction method in the factor analysis with varimax rotation. The sample consisted of (n = 27) teachers of basic general education of the Ibarra Canton.

**Results**: The results show a reliability analysis for the instrument obtained a Cronbach’s alpha (α = 0.901), estimated to be an excellent level.

**Conclusions**: The questionnaire is relevant, valid and reliable, adapting to the needs of teachers of basic general education to determine the nature or origin of the academic knowledge in a fast and reliable manner.

## Introduction

The concern and analysis about the knowledge possessed by teachers in general, and in particular those of basic general education, persists within the academic debate and in professional practice. ^1^^–^^3^ The nature of teachers’ education has become an important field of study considering the role it has in society.

In Ecuador, studies on the subject are scarce. For this reason, the need arose to carry out this research, with the intention of proposing a questionnaire to determine the origin of the academic knowledge of teachers of basic general education. In the bibliographic review, the absence of instruments to understand the configuration of the knowledge that a teacher puts into practice in the classroom was determined. From the perspective of this research, this fact is decisive for the quality of education.

In recent years, several studies have addressed the impact of teachers’ professional knowledge on the quality of teaching. There is a prevalence towards the analysis of pedagogical knowledge of the content, ^4^ which can be associated with a traditional conception of education.

At the same time, the academic community recognizes that a teacher’s level of knowledge is an indicator of the quality of their behaviour in the classroom. ^5^ This implies understanding this fact in terms of relational categories. It is worth mentioning that Shulman ^6^ was a precursor in considering that a teacher’s knowledge affects the student’s assessment of his capacity and with it of quality. This criterion becomes an important conceptual support for the analysis that occupies this work.

Additionally, it is striking that in the studies consulted, ^7^^,^^8^ there is no evidence that accurately determines the factors that make up the knowledge of a teacher, which function as support for the profession of teacher. It is possible that this is due to the multiplicity of ways in which knowledge is manifested and reached.

However, in mainstream literature, several authors have shown interest not only in the analysis of knowledge of the teacher, ^9^^–^^11^ but also in its implementation by designing tools that allow them to identify student´s learning styles and their pedagogical usefulness. ^12^^,^^13^

Building on this, it can be added that academic knowledge and its relationship with the teaching praxis are characterized firstly by being integrated into practice, which implies a continuous process of assimilation and organization of knowledge by the individuals involved. Secondly, they are situated within a temporal category that recognizes their emergence and evolution within a specific historical context and are related to a variety of knowledge. ^14^ The combination of theoretical and practical knowledge that a teacher possesses is a result of their academic training and professional experience in the corresponding field. ^15^

In other words, academic knowledge is considered an active, dynamic, and transformative relationship which is used to apprehend, understand and act on an environment. These relationships have the peculiarity of manifesting, reconfiguring and being open to debate. That is to say, these gradual development characteristics are the result of different processes, which are related to the teacher’s academic training in a specific disciplinary area, as well as their professional, affective, cultural, and social experiences that the teacher assumes.

Within this order of ideas, it should be considered that academic knowledge has been the center of attention when proposing new didactic strategies that the educator must consider according to the changes that are taking place in society. Likewise, several educational reforms that have been carried out from the point of view of teacher training take this knowledge as a starting point.

In Ecuador it is observed that in basic education, the role of the teacher is focused on the realization of micro-curricular plans and the incorporation of methodological strategies that involve the student at the center of the educational process. Additionally, it is noticeable that the teacher must develop appropriate resources and the types of evaluation to be carried out. Precisely, one of the aspects indicated to comply with the above comes from the combination of the teacher’s knowledge and professional skills. ^10^ To achieve this goal, they must achieve an innovation of educational work with flexibility in different contexts, as well as the exercise of a systematic pedagogical praxis.

It is also linked to certain performances developed by the teacher. Important knowledges are identified: to know the area of knowledge that teaches; to know how to teach the subject; to know how to teach in general; and to know how people learn.

The work “The seven necessary knowledges for the education of the future” by Edgar Morín ^16^ published by the United Nations Educational, Scientific and Cultural Organization (UNESCO), opened a space for reflection on education and the implications for the future of the quality of academic knowledge. In this regard, it has been possible to identify a variety of knowledge that teachers must possess ( Table 1).

**Table 1.** List of knowledge by authors.

Author, year	Knowledge
Morin, 1999	Recognizing the Blindness of Knowledge: Error and Illusion
	The principles of relevant knowledge
	Teaching planetary identity
	Facing uncertainties
	Teach ing understanding
	Ethics of mankind
Perafán, 2013	Academic knowledge
	Knowledge based on experience
	Knowledge based on routines and scripts
	Knowledge based on implicit theories
Gutierrez *et al*., 20 19	Learn to know
	Learn to do
	Learning to live together
	Learning to be
Ferrada *et al.*, 2021	Academic knowledge based on experience
	Academic knowledge based on implicit theories
	Academic knowledge based on scripts and routines
Aya-Villamil & Aristizábal, 2022	Curricular knowledge
	Disciplinary knowledge
	Experiential knowledge
	Pedagogical knowledge
De Gauna & Morán-Barrios, 2022	Know
	Know-how
	Knowing how to be

The aspects presented in Table 1 are based on the vision of reconfiguring the training of future teachers of basic education based on the paradigm shift of educational processes from teaching to learning. In this way, we proceed to solve those problems of praxis and those that directly affect the integral development of the student. This determines important adjustments not only of teachers, but of students and educational institutions. The former must act as true leaders and agents of change and transformation of didactic praxis. ^17^

Another important aspect to consider is given by the notions of implicit theories, ^18^ which configure the knowledge indicated. A review of several studies, ^19^ reports that teachers face difficulties in practice motivated by the way they perceive the teaching of a discipline, especially in the use of strategies loaded with values for which they are not prepared.

It shifts from focusing on what teachers know about the practice to their perceptions of its possibilities. This approach changes the understanding of the social context as an important influence on the way teachers make decisions about their practice. The role of previous experiences is fundamental in teachers’ perception of specific teaching situations. ^20^

The teachers’ perception of their practice refers to how teachers interpret and view their own work in the classroom; that is, how they understand and value their own performance in teaching. This includes the understanding of their role as educators, the pedagogical strategies they possess, their relationships with students, as well as their ability to respond to unforeseen situations, among other aspects. The teachers’ perception of their practice can influence their decisions and actions in the classroom, as well as their ability to improve and adapt to new situations.

A question arises before the proposal of this systematic construction carried out by teachers: why is it necessary to understand the experience of the profession, as well as the disciplinary structure of which it was formed and its concretion in practice? The answer may be oriented to the lack or weak mastery that this can exercise on knowledge of the subject, in addition to the curriculum and vocational training dictated or oriented in a fragmented and institutional way.

A second answer can be given by the specific disposition of the pedagogical situation through the educator that implies simplifications and exemplifications that allow learning on the part of the student. ^13^^,^^21^

Regardless of the historical period in which they were carried out, the studies do not respond to the need to break a pre-established disposition of disciplinary knowledge as a structuring mechanism of teaching and therefore of training. It is considered vital to reconfigure the vision that the teaching profession presents in practice.

Given these dilemmas and fragmentations, it is essential to determine the nature or origin of this knowledge oriented by personal identity, the work context and the experience accumulated by teachers.

These elements constitute an argument to conduct a new study that develops the construct of academic knowledge as a central theoretical core, which contributes to a better understanding of the factors that interact with the knowledge raised and that are expressed in the development of teaching practice.

Teachers need to identify academic knowledge to plan, teach and reflect effectively on instruction ^22^; for this purpose, measurement instruments are limited. ^23^ In the literature review, no studies or research were identified that analyse the knowledge of teachers in the conditions of the Ecuadorian territory and even more so in the educational units of the Ibarra Canton; nor the nature or origin of these.

It is recognized that there is a significant interest in determining the configurations of knowledge; however, evidence of its validity is still lacking. It is precisely this aspect that this proposal seeks to resolve. The objective of the study is to establish the construct validity and reliability of the questionnaire on the nature or origin of the academic knowledge of teachers of basic general education.

This knowledge has a broad, plural, integrating connotation, linked to social aspects. The proposed conceptual delimitation limits an academic knowledge closely linked to the exercise of reflection on knowledge. In this sense, basic education teachers do not limit themselves to teaching a pre-established curriculum, but develop their didactic activity based on practical knowledge, including their values and beliefs. ^24^

This last aspect is determined by macro and micro contextual factors, in addition to national educational policies and personal experiences. ^25^ In this way, the orientation of the present research towards the daily practices that interrelate praxis, the tradition of the trade and the disciplinary structure in which the teacher has been trained is established.

## Methods

### Study design

A methodological study was established with the objective of developing tests for validity, reliability and internal consistency processes. ^26^^,^^27^ The study consisted of education teachers working in educational units in the Ibarra Canton. The sample selection was convenience-based. ^28^^,^^29^

The study took place during the academic period from March to August 2022, starting in the fourth week of this cycle with three 45-minute training sessions. These sessions were conducted for 27 fifth-semester students who were undertaking their pre-professional internships in 27 educational units in the Ibarra Canton as part of the project.

In the fifth week, the researchers contacted 27 Basic Education teachers, with prior approval from the authorities of their educational units. In this action, the students played a fundamental role in facilitating closer contact with the institutions. Immediately, the “Questionnaire on the nature or origin of academic knowledge of Basic Education teachers” was distributed to all teachers, with the objective of determining its validity, reliability, and internal consistency.

Although the educational units were selected by non-probabilistic methods, the data presented ^30^ in their study show a high correspondence in the results between nationally representative samples and convenience samples at the local level. Therefore, it is considered valid to use this type of sample for the current research.

As inclusion criteria, the level of teaching of the teachers, specifically Basic Education, was established, as well as availability to participate in the study. The teachers participated voluntarily, giving their informed consent to ensure confidentiality and anonymity of the participants. In addition, the confidentiality of the data was maintained by storing and processing them in an exclusive database with access only by the authors of the study. Expert judgment was used to determine the validity of the instrument.

All work was approved by the Honorable Board of Directors of the Faculty of Education, Science, and Technology of the Universidad Técnica del Norte (Resolution No. 239-2021-HCD). Approval for the study was dated March 26, 2022, as part of the research project: Didactic Transposition and Academic Knowledge of Basic Education Teachers in the Ibarra Canton. This work was carried out in accordance with the guidelines of the university’s code of ethics. ^31^ Finally, each participating educational unit was provided with a report containing information on the overall results of their unit as well as the total sample. This report explains the most significant findings as well as future guidelines for action based on scientific evidence, based on the results obtained.

### Procedure

The literature review was carried out considering the following assumed research question: How to establish the construct validity and reliability of the questionnaire on the nature or origin of academic knowledge of basic education teachers? The identified keywords for the search were: construct validity and reliability, academic knowledge of teachers, basic education.

The inclusion criteria were: 1) studies focused on basic education; 2) studies in education that report the construction of questionnaires; 3) empirical or primary studies; 4) studies in French, English, Portuguese, and Spanish. The aspects considered for exclusion generally reflected studies focused on populations other than basic education or that did not include teachers in their samples. Additionally, other analyses that showed viewpoints and opinions, such as editorials, letters to the editor, comments, or perspectives, were excluded.

To meet these criteria, a search was conducted in the databases Web of Science, Scopus, Taylor & Francis, Science Direct, Springer, and Scielo. As a result of the initial analysis, documents that did not meet the inclusion criteria were eliminated, including studies focused on populations other than basic education or that did not include teachers in their samples. In addition, other analyses that showed points of view and opinions, such as editorials, letters to the editor, commentaries or perspectives, were excluded. In addition, the search was complemented with a manual review of the most relevant journals on the subject.

From the total number of identified articles, duplicate articles were eliminated, then a selection was made considering the title, abstract, and finally reviewing the full text, thus eliminating irrelevant research based on the inclusion and exclusion criteria. From the remaining articles, a backward citation searching was performed. This technique was especially useful in finding classic or fundamental studies in the field of quantitative studies in education.

Once the final number of selected articles was obtained, they were exported to the reference manager Zotero. The process of selecting articles was carried out independently by two researchers, with the mediation of a third researcher in case of differences of opinion.

The questionnaire is an adaptation of the Pedagogical Content Knowledge (PCK) Competence Model for physics teachers in initial training. ^32^ Several steps were defined for the adaptation process of the questionnaire.

The first step involved an initial translation into Spanish by the authors of this research, which was later reviewed by subject matter experts. Then, the original questionnaire was evaluated to determine its suitability for the target population and context. The referenced model is based on the refined consensus of PCK, which is defined as a set of knowledge that teachers need to teach their subject matter in a way that is understandable for students. The authors identified three essential components of PCK: 1. Content Knowledge, 2. Pedagogical Knowledge, and 3. Pedagogical Content Knowledge.

Subsequently, in order to identify the understanding of the translated version of the questionnaire, it was sent to a group of judges (n=8), who were experts in the field of education. The selection criteria were people who had graduated in Basic Education and were working in educational units in Cantón Ibarra. This was done to evaluate the quality of the translation and ensure that it was accurate and culturally appropriate.

The questionnaire was then adjusted to make it relevant and appropriate to the culture of the target population and to meet the proposed objective. After this initial analysis, one team member proposed the first draft of the instrument. It had four variables with a total of 17 indicators: Disciplinary Knowledge (5 indicators), Experience-Based Knowledge (4 indicators), Theory-Based Knowledge (3 indicators), and Script and Routine-Based Knowledge (5 indicators).

The first draft, agreed upon by the other team members, underwent validation to ensure that it was valid and reliable for the target population. The process was carried out by the expert judges (n=8).

Once this validation process was completed, one team member drafted the second version of the instrument. It was based on the percentage of agreement obtained in the assignment of scores, and the wording of those where at least 80% agreement had not been reached was modified. A second version of the questionnaire with seven items and a high degree of agreement was obtained. The new proposal had eliminated one variable (Script and Routine-Based Knowledge: 5 indicators). Additionally, several indicators from each of the variables were excluded: Disciplinary Knowledge (3) and Theory-Based Knowledge (2).

The proposal was discussed and accepted. The final version of the instrument, “Questionnaire on the nature or origin of academic knowledge of elementary education teachers,” consists of seven indicators that respond to three variables. The suggested response scale is a Likert-type scale, ^34^^,^^35^ with five levels that range from Completely Disagree (1), Disagree (2), Neither Agree nor Disagree (3), Agree (4), and Completely Agree (5).

The first phase involved estimating the content validity ^36^^,^^37^ based on the assessment of expert judges (n=8) in the field of education. ^38^^,^^39^ These judges were selected and contacted from a list of teachers who act as supervisors for pre-professional practices in different educational units where students of the Basic Education career collaborate. This requires a mastery of institutional educational development and teacher training in particular. They hold fourth-level degrees in the specialties of Curriculum Design, Educational Technology, and Educational Innovation. None of them are associated with the researchers of this study, so they express no conflict of interest.

These expert judges were provided with a guide to the objectives of the study and the second draft of the instrument virtually. They were asked to rate each item in terms of sufficiency, clarity, and coherence. Each indicator was scored using a scale with 1 (Totally Disagree), 3 (Neither Agree nor Disagree), and 5 (Totally Agree). The kappa index, ^40^ was applied to measure the stability of the tool. It was weighted for the established indicators (standard: > 0.41: moderate).

The second phase focused on validating the “Questionnaire on the nature or origin of academic knowledge of basic education teachers” through the determination of its validity, reliability and internal consistency by the general basic education teachers who participated in the research (n=27).

### Data analysis

The obtained results were loaded into Excel (version 26.0) and incorporated into the Statistical Package for Social Sciences (SPSS) package (version 25.0) for descriptive and inferential analysis.

An exploratory factorial analysis, ^41^ was performed on principal components using the varimax rotation method. Indicators with communalities >0.3 were considered valid. The internal consistency of the entire instrument was estimated using the Cronbach’s alpha coefficient, with a minimum acceptable value of (α≥0.70) estimated.

## Results

Of the 27 teachers of basic general education who participated in the research, 81% were female and 19% were male. The median age was 40 years (range: 24-57 years). An important factor has to do with years of teaching experience, which averages 12.37 years (range: 1-30 years). 68% of teachers have degrees in Education Sciences.

Table 2 reports the knowledge of the teachers. Table 3 reports the reliability between the different evaluators (Cohen’s Kappa) (n=8), where the result obtained was substantial (range: κ=0.61- κ=0.80) for two aspects (sufficiency and clarity) and moderate (range: κ=0.41- κ=0.60) for the remaining (coherence). The sufficiency has obtained the highest values, allowing us to consider that the different indicators belong to the same dimension considered as the academic knowledge of the teachers.

**Table 2.** List of knowledge and indicators on the nature or origin of the academic knowledge of teachers of basic general education.

Code	Variables	Indicators
Var01	Disciplinary knowledge	In the preparation of categories, concepts definitions, topics, contents I teach; I pay more attention to the knowledge of the disciplines I teach than to their didactic treatment
Var02	Disciplinary knowledge	He evaluated the concepts of everyday life and in this way structured the contents that must be taken into account for the development of the different disciplines
Var03	Knowledge based on experience	My life experiences and personal stories are sources that have contributed to the construction of the categories, concepts, definitions, themes, contents that I develop as a teacher
Var04	Knowledge based on experience	The development of my professional life has changed as a result of the relationship with students, colleagues and other actors outside the school system.
Var05	Knowledge based on experience	I am used to systematically reflecting on the knowledge of the discipline I teach linked to the teaching-learning processes
Var06	Knowledge based on experience	In the exercise of reflection that I carry out on the professional practice as a teacher I produce some kind of knowledge or knowledge
Var07	Knowledge based on implicit theories	School and institutional regulations have played a fundamental role in the construction of the categories, concepts, definitions, themes, contents that I develop as a teacher.

**Table 3.** Reliability score among evaluators by aspect.

Aspect	Cohen's K	P value	Degree of agreement
Sufficiency	0.73	0.000	Substantial
Clarity	0.71	0.000	Substantial
Coherence	0.55	0.000	Moderate

As for the descriptive analysis, it presents an average response that goes between 4 and 5, which assigns values between “Agree” and “Totally agree”, where 87% belongs to the latter value ( Table 4).

**Table 4.** Summary descriptive analysis by questionnaire indicator for (n=27).

Codes	Valid		Frequency	Percentage	Valid percentage	Cumulative percentage
Var01	Valid	4	3	11.1	11.1	11.1
		5	24	88.9	88.9	88.9
Var02	Valid	4	3	11.1	11.1	11.1
		5	24	88.9	88.9	100.0
Var03	Valid	4	4	14.8	14.8	14.8
		5	23	85.2	85.2	100.0
Var04	Valid	4	5	18.5	18.5	18.5
		5	22	81.5	81.5	100.0
Var05	Valid	4	5	18.5	18.5	18.5
		5	22	81.5	81.5	100,0
Var06	Valid	4	3	11.1	11.1	11.1
		5	24	88.9	88.9	100.0
Var07	Valid	4	2	7.4	7.4	7,4
		5	25	92.6	92.6	100.0

Table 5 presents the analysis of global reliability of the instrument. Cronbach’s alpha is 0.901, and it is estimated as an excellent level. ^42^ For the principal components analysis, a validation analysis of assumptions was developed using the Bartlett statistic and the KMO value. Bartlett’s sphericity test was used to show if there were significant correlations in the data. The values <0.05 support that the technique is adequate (165,591; gl=21; p<0.000), ^43^ the model is appropriate and does not present sphericity. The KMO sample adequacy measure was 0.778, which is considered medium.

**Table 5.** Summary analysis for reliability and value of commonalities.

Codes	Dev. Deviation	Cronbach's alpha if the element has been deleted	Extraction
Var01	0.32026	0.896	0.914
Var02	0.32026	0.870	0.841
Var03	0.36201	0.866	0.961
Var04	0.39585	0.871	0.833
Var05	0.39585	0.916	0.615
Var06	0.32026	0.896	0.736
Var07	0.26688	0.880	0.891

It was considered that the recommended ratio of commonalities should be ≥ 0.40. ^44^ Of the 7 indicators that are part of the questionnaire, all are considered useful, and have adequate factorial weights. The interval obtained was (=6.15 and =0.96) ( Table 5).

The results of the application of the principal components extraction method in the factor analysis with varix rotation indicate the retention of two factors for presenting an eigenvalue greater than 1 ^45^; see Table 6.

**Table 6.** Results of the application of the principal component extraction method in factor analysis with varix rotation.

Total variance explained						
Initial eigenvalues				Sums of loads squared of extraction		
Indicators	Total	% Variance	% Cumulative	Total	% Variance	% Cumulative
1	4.570	65.283	65.283	4.570	65.283	65.283
2	1.222	17.458	82.741	1.222	17.458	82.741
3	0.645	9.219	91.960			
4	0.247	3.534	95.494			
5	0.159	2.268	97.762			
6	0.112	1.600	99.361			
7	0.045	0.639	100.000			

Extraction method: principal component analysis.

**Figure 1.**
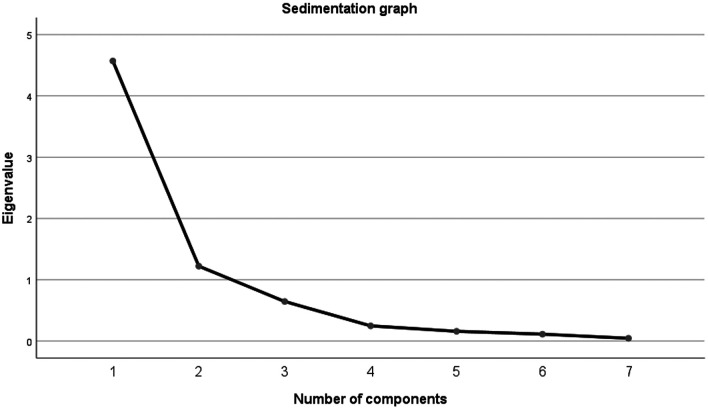
Scatter plot of each of the factors.

When considering both, an explained variance of 82.74% is obtained. Figure 1 presents the eigenvalue scatter plot of each of the factors; 2 factors have eigenvalues higher than (>1).

Thus, the first indicator called “In the preparation of the categories I pay more attention to the knowledge of the disciplines” that retains 65.28%; and a second indicator “I evaluate the concepts of everyday life and in this way I structure the contents” with 17.46%.

## Discussion and conclusions

The possibilities of academic knowledge in education have not been fully explored. ^45^ None of the previous studies has yet explored this knowledge, and the different configurations that support them. Although other studies have addressed the issue, ^46^^,^^47^ they have only done so from the perspective of teachers’ content knowledge. That is, the studies have focused particularly on teachers’ professional knowledge for effective teaching. ^45^

The original instrument from which this questionnaire was adapted was an important measure that can be used to determine the nature or origin of teachers’ academic knowledge. It includes several indicators that in the context of Ecuadorian basic general education have not been recognized or valued by experts previously. Previous studies have described the importance of teachers’ application of their knowledge in the classroom, which allows complex reasoning processes to be involved, selectively identifying those most relevant, from their perspective, to put them in context in a particular situation. ^48^

However, the relationship of the implicit theories of the teachers, in addition to the different ways that they teach their classes, have been ignored or not understood their true consequence in didactic practice. The proposal presented also shows differences with respect to the “competence model for teachers’ pedagogical content knowledge.”. ^49^ The reliability analysis obtained an excellent level. These values are consistent with, ^50^ authors who in their research obtained (α=0.981), with values greater than (α=0.80) in all indicators. This reveals a good internal consistency of the instrument, similar to Monroy. ^49^

In line with Şimşek, ^49^ the idea of considering academic knowledge as a coherent and plausible construct is supported. This makes it possible for the validated questionnaire to become an excellent candidate to identify the nature or origin of the academic knowledge of teachers of basic general education without the need to reduce or eliminate indicators from the instrument. ^51^ Currently, there are few scales that meet these conditions: fast administration, no need to delete items, and high values of Cronbach’s alpha. It is shown that the instrument has high values of internal consistency, measured by Cronbach’s coefficient, as well as by its reliability. ^52^ The scale presented constitutes a reliable tool, ^53^ to assess the nature or origin of the academic knowledge of teachers of general basic education.

According to the results of the exploratory factor analysis, the Kaiser-Meyer-Olkin value fits with other studies conducted in the area of education. ^49^^,^^54^ In some of these, ^55^ different items were removed from the scales for a factorial load value less than 0.50; however, in the current analysis the values obtained are above the standardized threshold, ^56^ indicating that the correlations between questions are statistically significant and sufficiently strong ^57^ for the analysis of questions. These considerations are strengthened by studies that support the presence of values on a scale between =0.70 and =0.95. ^58^ The principal components analysis using the varimax rotation method to explore the factorial structure of the teachers’ academic knowledge scale was based on the criterion (eigenvalue>1). ^59^ From the 7 initial indicators the procedure of reduction. That is, the questionnaire on the nature or origin of academic knowledge of teachers of basic general education represents a robust and valid framework to understand certain notions of implicit theories and the role of experiences as the basis and support of academic knowledge. In reference to the different indicators that make up the scale, they have adequate factorial weights and no item presented commonality <0.55 (ranges=0.553 and =0.795). ^60^ This has been supported by published and reviewed studies. ^61^^,^^62^ which demonstrate the usefulness and saturation of the scale through its different items in order to constitute a theoretical construct and that it allows the identification of the nature or origin of the academic knowledge of teachers quickly and reliably with an instrument composed of 7 indicators.

The present study examined the validity and reliability of a scale on the nature or origin of the academic knowledge of teachers of basic general education, which can facilitate research in this field and provides a conceptual framework to understand certain notions of implicit theories and the role of experiences as the basis and sustenance of academic knowledge. The ‘Questionnaire on the nature or origin of academic knowledge of teachers of basic general education’ presents an advantage over other instruments since it focuses on the beliefs and implicit theories that are part of the teacher’s thinking. To this end, it determines the role played by disciplinary knowledge and its didactic treatment; the definition of the sources that contribute most to the teaching exercise. In addition, it can demonstrate reflection and change during their professional life.

The levels of reliability between the different evaluators show an excellent intraobserver correlation. Evidence related to reliability analysis was collected for the entire instrument with a Cronbach’s alpha value (α=0.901), considering the 7 indicators, which is estimated as an excellent level. Bartlett’s sphericity test showed that, if there are significant correlations in the data, this supports that the technique is adequate and that the model is appropriate and does not present sphericity. Of the 7 indicators that are part of the questionnaire, all are considered useful and have adequate factorial weights. The usefulness and saturation of each of these to constitute the theoretical construct related to the academic knowledge of teachers of basic general education is demonstrated. This constitutes a relevant, valid and reliable instrument to determine the nature or origin of the academic knowledge of teachers in a fast and reliable manner.

The present study has some limitations, which are not related to the fulfilment of the proposed objective; however, it is necessary to mention them. The first is related to the absence of other questionnaires as a standard with which to compare the results obtained. We have reported on the non-existence of measures that serve as a standard. In light of this, the strategy adopted was based on the review of bibliographic material that reflected studies on the construction of similar tools from the methodological point of view and their application to education. A second limitation of the present study considers that the extrapolation of the results obtained for use in other educational units that did not participate in the sample is based on the participation of (n=27), teachers from the same number of institutions. However, this can be minimized considering the responsibility.

The Questionnaire on the Nature or Origin of Academic Knowledge of Basic General Education Teachers focuses on the implicit beliefs and theories of basic education teachers. It determines the role of disciplinary knowledge and its didactic treatment, as well as the sources that contribute the most to teaching practice and reflection during their professional life. The reliability test showed that the questionnaire is acceptable with a Cronbach’s alpha of =0.704, standard deviation values between =0.76 and =0.97, and appropriate factorial weights for each item. The questionnaire is relevant, valid, and reliable, tailored to the needs of basic general education teachers, and confirming the construct validity and reliability of the questionnaire.

### Ethical considerations

The entire study was approved by the Honourable Board of Directors of the Faculty of Education, Science and Technology of the Technical University of the North (Resolution No. 239-2021-HCD), as part of the research project: Didactic transposition and academic knowledge of teachers of basic general education of the Ibarra canton. This work was carried out in accordance with the guidelines of the university code of ethics 2012. Those who voluntarily decided to collaborate in this research signed a written informed consent to ensure the confidentiality and anonymity of the participants.

## Data Availability

Open Science Framework: Validity and reliability of the questionnaire, https://doi.org/10.17605/OSF.IO/WBNKC. ^63^ This project contains the following underlying data:
- Data_knowledge.sav contains the primary data of the sample that participated in the study (27 basic education teachers) related to gender, age and years of experience, as well as the assessment of the 7 items of the “Questionnaire on the nature or origin of the academic knowledge of basic general education teachers”. - Data_judges.sav contains the primary data of evaluations by experts (n=8) on the indicators of adequacy, clarity, and coherence of the questionnaire. This served as a basis to determine the reliability among the different evaluators (Cohen’s Kappa). Open Science Framework: Validity and reliability of the questionnaire, https://doi.org/10.17605/OSF.IO/WBNKC. ^63^ This project contains the following underlying data:
- Results_knowledge.htm, contains the findings from the analysis and comparison of various statistics necessary to fulfil the objective of testing the validity, reliability, and internal consistency processes, as assessed by the study participants. - Results_knowledge.spv contains the SPSS output. - Instrument.pdf, the instrument that was given to teachers for validation. -
Table 2. List of knowledge and indicators.xlsx Data are available under the terms of the Creative Commons Zero “No rights reserved” data waiver (CC0 1.0 Public domain dedication).
