# Supplementary material for: Validity and reliability of the questionnaire of academic knowledge of teachers of basic general education
Source: F1000Res. 2023 Jun 12;12:642. [Version 1] doi: 10.12688/f1000research.134261.1 (PMC11112302; doi:10.12688/f1000research.134261.1)
Supplement: Peer review history

# Reviewer report: Reviewer response for version 1

**DOI:** 10.5256/f1000research.147302.r212266

**Published:** 2024-05-22

**Authors:** Laura Guerra

**Affiliations:** 1 Pontificia Universidad Catolica del Ecuador, Quito, Pichincha, Ecuador

Why did you say that “The sample selection was convenience-based”? Please, explain this point more.

 The period considered for the review of the literature is not mentioned.

 All the articles reviewed coincided with the constructs/variables considered in the questionnaire chosen to translate and adapt it to your research? You said that the questionnaire is an adaptation of the Pedagogical Content Knowledge (PCK) Competence Model for physics teachers in initial training.

 I understand that 27 basic education institutions in Ibarra were considered. Therefore, it is not clear if you worked with only one professor from each institution. It was presented in the results, institutions = teachers?

Is the work clearly and accurately presented and does it cite the current literature?

Yes

If applicable, is the statistical analysis and its interpretation appropriate?

Yes

Are all the source data underlying the results available to ensure full reproducibility?

Yes

Is the study design appropriate and is the work technically sound?

Yes

Are the conclusions drawn adequately supported by the results?

Yes

Are sufficient details of methods and analysis provided to allow replication by others?

Partly

Reviewer Expertise:

Technology, education and society

I confirm that I have read this submission and believe that I have an appropriate level of expertise to confirm that it is of an acceptable scientific standard.

# Reviewer report: Reviewer response for version 1

**DOI:** 10.5256/f1000research.147302.r259132

**Published:** 2024-04-22

**Authors:** Kezang Sherab

**Affiliations:** 1 Paro College of Education, Royal University of Bhutan, Thimphu, Thimphu, Bhutan

This manuscript reports a research on testing the validity and reliability of the questionnaire on academic knowledge of basic general education teachers in Ecuador. The study was mainly focused on establishing the construct validity and reliability of the questionnaire designed to check the nature or origin of the academic knowledge of the basic general education teachers. The study conducted with 27 basic general education teachers showed excellent reliability of the questionnaire and the authors suggest it to be used to determine the nature or origin of the academic knowledge. 

 The literature used are fairly recent and the data analytical approach used is quite robust. However, there are a few minor comments for the authors to consider.

 First, the title could be rephrased to avoid some of the 'of'. Currently there are four 'of' in the title.

 Secondly, the term 'teachers of basic general education' may not be understood by all readers, especially from other contexts. So it would be useful for the authors to make this term clear with appropriate explanation right from the introduction section.

 Thirdly, it has been observed that the Cronbach's Alpha level is reported in three decimal places. As far as I am concerned, it should be reported in two decimal places. Similarly, in Table 5 standard deviation should be reported in two decimal places. 

 Fourthly, In the title of Table 6, the phrase varix rotation should be varimax rotation I think.

 Lastly, the limitations of the study are presented along with the discussion and conclusion section, thought it would provide clarity if the limitations of the study is presented in a separate section.

Is the work clearly and accurately presented and does it cite the current literature?

Yes

If applicable, is the statistical analysis and its interpretation appropriate?

Yes

Are all the source data underlying the results available to ensure full reproducibility?

Yes

Is the study design appropriate and is the work technically sound?

Yes

Are the conclusions drawn adequately supported by the results?

Yes

Are sufficient details of methods and analysis provided to allow replication by others?

Yes

Reviewer Expertise:

teacher education, non-cognitive skills, student engagement, TPACK, values education,

I confirm that I have read this submission and believe that I have an appropriate level of expertise to confirm that it is of an acceptable scientific standard.
